# The library is open: a scoping review on queer representation in psychedelic research

**DOI:** 10.3389/fpubh.2024.1472559

**Published:** 2024-12-11

**Authors:** Amy Bartlett, Challian Christ, Bradford Martins, Kellen Saxberg, Terence H. W. Ching

**Affiliations:** ^1^Department of Classics and Religious Studies, University of Ottawa, Ottawa, ON, Canada; ^2^Department of Psychiatry, Yale University School of Medicine, New Haven, CT, United States; ^3^School of Psychology, University of Ottawa, Ottawa, ON, Canada

**Keywords:** queer, psychedelics, mental health, scoping review, LGBTQ, gender, sexuality, identity

## Abstract

The intersection of queer identity and psychedelics has not been thoroughly explored by the research community, historically or in the present day. With growing access to legal psychedelic therapies, it is essential that queer psychedelic experiences are understood sufficiently by clinicians in order to provide the most safe and effective care possible. Psychedelics and queerness are intricately related, and there is strong interest in the use of psychedelics for healing and identity development among queer populations. However, the vast majority of the literature stigmatizes and problematizes queer psychedelic use. Therefore this scoping review seeks to explore the current and historical overlap between psychedelics and queerness in the academic literature. Specifically, this scoping review aims to understand the available academic literatures’ treatment of the meaningful, non-pathologizing use of psychedelics within the queer community, and seeks to highlight the unique potential a queer lens and the queer experience can bring to the study of psychedelics. To do so, we asked what queer psychedelic experiences are reflected in the literature, who is being studied, what queer individuals’ motivations are for using psychedelics, and a review of the impacts of queer psychedelic use discussed in the literature. Literature searches were performed in seven academic databases using a wide breadth of both queer-related and psychedelic-related keywords, which resulted in over thirty thousand resources being captured. After screening, a total of 18 resources were collected as representative of the meaningful overlap of psychedelics and queerness. Based on the findings and research gaps identified, this scoping review makes several recommendations regarding future directions psychedelic researchers and clinicians can pursue to better understand and benefit from the meaningful overlap of psychedelics and queerness. By reclaiming, redefining, and reimagining the meaningful relationship between psychedelics and the queer experience, this review helps move the scientific and clinical conversation into queerer spaces, centering queerness and queer experiences as an essential component of psychedelic research and practice.

## Introduction

Non-ordinary states of consciousness have been accessed by humans for millennia through a variety of material and experiential means. Psychedelic substances are one such avenue for accessing non-ordinary states, and have long been intertwined with the human experience (1). Substances such as psilocybin, lysergic acid diethylamide (LSD), peyote, ayahuasca/dimethyltryptamine (DMT), and 3,4-methylenedioxymethamphetamine (MDMA) offer users a range of experiences, perspectives and psychological shifts that grapple with some of the most fundamental questions around what it means to be human (2, 3). A growing body of clinical research suggests such experiences can be harnessed therapeutically to aid in the treatment of substance use disorders (4–6), depression (7–9), anxiety (10), and end-of-life distress (11, 12). This, in turn, has increased interest in the phenomenology and impact of psychedelics in recent academic discourse.

In parallel with the renewed interest in psychedelic influence on mental health, spiritual exploration, and human flourishing, so too is there a broad and evolving conversation around identity. Sexuality, gender identity and queerness^1^ play a pivotal role in shaping the framework of individual identity (13). Increasing research has shown that holistic care of an individual, including sexuality, in clinical settings improves treatment outcomes and retention (14). While the relationship between health and sexuality has historically focused on greater disease burden and risk exposure (15–17), many clinical groups are now implementing training in LGBTQ+ affirming care to increase positive health outcomes in this population (18, 19). When individual identity is given more consideration in clinical care therapeutic interventions reach underserved populations in greater numbers, as is evidenced by the disparity in PrEP (Pre-Exposure Prophylaxis) usage between individuals whose sexual identity was affirmed during their care and those who were not despite both groups having access to the treatment (20). As the field continues to affirm the therapeutic potential of psychedelics, the relationship between queer identity and psychedelic use has not been thoroughly explored by mainstream researchers, historically, or in the present day. This gap in research at the intersection of queer identity and psychedelics limits the possible implementation of equitable clinical policies and procedures in psychedelic therapy, and further marginalizes this group of people already underserved by healthcare systems.

As the field of psychedelic studies continues to develop psychedelic-assisted treatments for a range of mental health issues (21) and develop a more comprehensive picture of the spiritual, religious and cultural relevance of these substances, it is imperative to establish an inclusive orientation to the study of psychedelics that meaningfully integrates individual gender and sexual identity into our understanding of psychedelic experiencing. The current trend toward the medicalization of psychedelic use has resulted in a limited discussion around the contributions of spiritual and personal use in support of the development of self-identity and self-acceptance (22). While these conversations are occurring in selected spaces (23), there has to date been no review of the literature on psychedelic use and queer lived experience. Furthermore, while considerations of gender and sexual identity have been included in psychedelic research, these have been superficial and tokenistic at best, and pathologizing and stigmatizing at worst.

The number of people who identify as queer has increased, with an average of 9% of the population across 30 countries now identifying as lesbian, gay, bisexual, or trans (LGBT) (24). Despite growing numbers of people identifying as queer, ‘coming out’ is still often met with violent opposition. As a result, most suicide attempts of queer individuals occur within the first 5 years of realizing one’s sexual identity, irrespective of age; though for many, this is during their youth (25). More than half of U.S. trans and non-binary people aged 13–24 considered killing themselves in 2023, while queer teens attempt suicide at a rate more than twice that of their straight peers (26). During the process of understanding one’s identity in a society where some (often many) people oppose your very existence the ideal of self-acceptance is often unattainable and self-actualization unfathomable. Given their potential to help treat depression (27) and existential dread (28) queer psychedelic use in a non-stigmatizing and non-pathological manner may be an under-researched avenue for improving patient care and preserving queer lives.

We contend that psychedelics are inherently queer. Psychedelic use has been interwoven in Indigenous societies with fluid conceptions of gender and sexuality (29). Psychedelics have been shown to release rigid patterns, make new connections, invert expectations, and challenge societal norms (30). From found family and community in queer spaces to the therapeutic care of long-neglected parts of oneself, psychedelics have the potential to promote queer healing and acceptance. The Pride flag, now recognized across the world as a symbol of community, was conceived of during an acid trip in the era when queer people found community and catharsis on the dance floor using MDMA and LSD [(31), p. 61]. Even the fungal kingdom from which psilocybin is derived is queer, with one species of fungi having more than 23,000 distinct sexual identities (32). As mycologist Merlin Sheldrake (33) observes, this helps scientists think beyond the binary, mirroring queer theory and reflecting the world in its crystalline multiplicity (pp. 90-91).

The primary aim of this scoping review is to better understand the current and historical discourse occurring at the intersection of psychedelics and queerness in academic literature. Until fairly recently the narratives of both psychedelic use and queer identity have been controversial, if not stigmatized entirely. From LSD being used in conversion therapy (34) to the stigmatization and pathologization of MDMA use among gay men in the 1980s-2000s (35, 36), the intersection of queerness and psychedelics has had a complicated history. This historical context is further muddied by the limited research and publication from a queer lens on psychedelic use within the queer community.

Part of the approach taken in this scoping review was to maintain a healthy skepticism of the heteronormative approach to research, including questioning the dominant research ideological framework and methodology, both historically and in modern research. With this framework in mind, the review aims to emphasize the concept of non-pathologizing, psychedelic use within the queer community. The authors of this manuscript, all of whom identify as members of the queer community, wanted the focus of this review to forgo including literature that further perpetuates negative stereotypes or stigmatization of psychedelic use and queer individuals. The review instead aims to highlight the unique potential a queer lens and the queer experience can bring to the study of psychedelics, as well as the potential for psychedelics to meaningfully support queer self-identity. To these ends, the group defined resources that contained a ‘meaningful’ overlap of queer lived experience and psychedelic use as:

- First-hand accounts of psychedelic use from queer identifying individuals
- Studies on psychedelic use performed through a queer lens that either sought to critique heteronormative exclusivity in research or benefit the queer community
- Accounts of historical use, whose content while potentially problematic, were not written with intent of further stigmatization of or violence toward the queer community
- Opinion pieces aimed at empowering queerness

Guided by this orientation, the paper thus seeks to review the current state of academic literature surrounding the meaningful intersection of queerness and psychedelics. We begin by outlining the methods undertaken for this scoping review, followed by a qualitative analysis of the results and an exploration of the emerging trends within our sample. Our initial research questions were:

1. What queer psychedelic experiences (e.g., types of psychedelics, settings of use) are captured in the studies screened in?
2. Who is being studied?
3. Why do queer people use psychedelics?
4. What are the impacts of queer psychedelic use discussed?
5. Based on these findings, what trends and gaps can be identified in the discourse on queer psychedelic use, and where should future research focus?

## Methods

### Search strategy

Literature searches were performed in the following databases: PsycInfo; MEDLINE; GenderWatch; Sociological Abstracts; PQ dissertations & Theses Global; Scopus; and Web of Science. In order to create a comprehensive capture of all relevant publications, we did not specify a start date in these searches. The initial search was conducted by SMM and CC in September 2020. An updated search was undertaken in May 2023 by CC and KS to capture any publications that had been published in the interim (i.e., between October 2020 and May 2023).

The following keywords were used in both search phrases to capture queer-related resources: queer*, gender*, genderqueer, trans*, two-spirit, homosex*, nonheterosex*, pansex*, sexuality, sexual minorit*, sexual orientation, LGB*, gay, lesbian, questioning, men who have sex with men, MSM, women who have sex with women, WSW. The following keywords were used in both searches to capture psychedelic-related resources: psychedelic*, hallucinogen*, entheogen*, tryptamine, phenethylamine, LSD, lysergic acid diethylamide, psiloc*, magic mushroom*, DMT, N,N-Dimethyltryptamine, 5-MeO-DMT, ayahuasca, 3,4,5-trimethoxyphenethylamine, ecstasy, MDMA, dimethyltryptamine, Banisteropsis caapi,” psychotria viridis, mescaline, peyot*, serotonergic, 5HT2A, microdos*. In order to capture publications which discussed *both* queerness and psychedelics, publications with at least one keyword belonging to both the queer-related keywords pool and the psychedelic-related keywords pool were captured and screened.

### Selection criteria

Selection criteria were established ahead of the extensive screening process, and were shaped by a critical, queer-affirming approach to showcase meaningful, non-stigmatizing psychedelic publications. Publications were screened in for deeper review if there was a meaningful, non-stigmatizing overlap between LGBTQ+ issues *and* psychedelics (e.g., non-stigmatizing analysis or discussions of psychedelic use by LGBTQ+ individuals in ways that center queer lived experiences). Publications were excluded if they: (1) did not discuss LGBTQ+ issues; (2) did not discuss psychedelic experiences; (3) focused only on prevalence of psychedelic use in LGBTQ+ communities; (4) discussed psychedelic use by LGBTQ+ individuals in a solely stigmatizing way; or (5) merely mentioned psychedelic use by LGBTQ+ individuals without further discussion or analysis.

## Results

**Figure 1:**
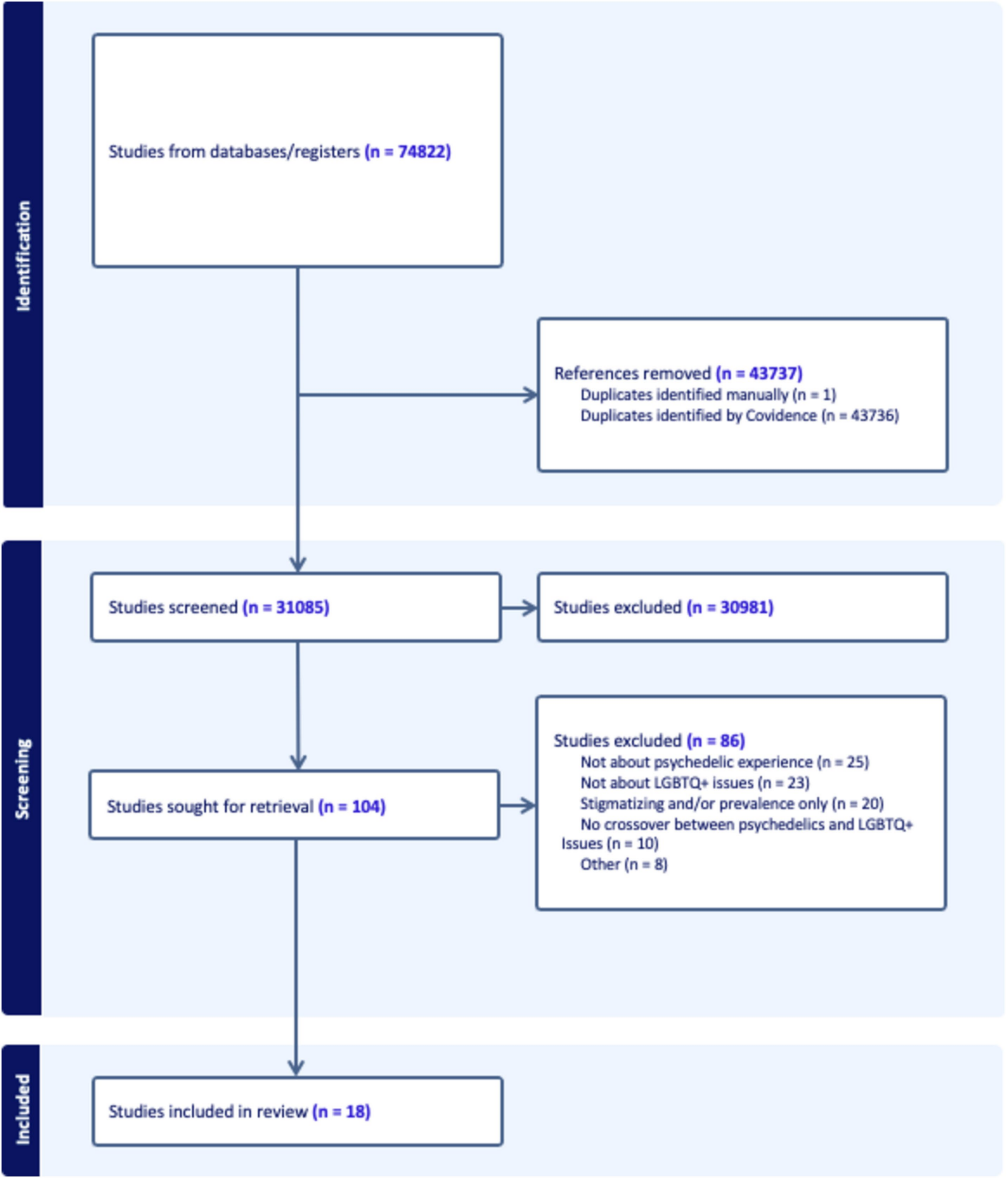
Covidence screening flowchart.

In total, 74,822 articles with 43,737 duplicates were captured for screening in the Covidence platform. Of the 31,085 publications screened, 30,981 publications were screened out due to their irrelevance, leaving 104 publications to be assessed for potential inclusion eligibility. Of these, 86 were identified by at least two reviewers as meeting exclusion criteria and were removed following discussion with all reviewers. It was determined that 25 sources did not discuss psychedelic experience; 23 did not discuss queer issues; 20 were unilaterally stigmatizing of psychedelic use among queer populations or concerned only with prevalence of drug use in queer populations; 10 failed to discuss how queer issues related to psychedelic experiences; and 8 were excluded due to being published in a language the authors did not speak, or because they did not address our research questions (see Figure 1).

The remaining 18 publications met the selection criteria and were extracted for review (see Table 1). Each extracted publication was systematically reviewed and annotated independently by two reviewers to ensure inter-rater reliability. Discrepancies in annotation were resolved within each reviewer pair and with the larger reviewing team via a consensus decision-making approach (37).

**Table 1:** Studies screened in.

Author & Year	Location of Study	Study design	Psychedelic substance(s) used/discussed	Sample Size	Mean Study participant age (range)	Study participant gender breakdown	Study participants sexual identities breakdown
Agin-Liebes et al., 2021	USA	Clinical trial; Qualitative	Psilocybin	*n* = 9	57.9 (50–64)	Cis-Male (100%)	Gay (100%)
Alpert, 1969	USA	Case study; Qualitative	LSD	*n* = 1	38	Cis-Male (100%)	Bisexual (100%)
Anderson et al., 2020	USA	Experimental	Psilocybin	*n* = 18	59.2 (50–66)	Cis-male (100%)	Gay (100%)
Bryant, 2016	Canada	Qualitative	All psychedelics	*n* = 5	35.6 (30–40)	Cis-female (40%) Cis-male (20%) Two-spirit (20%) Non-binary (20%)	Queer (100%) [Open-ended descriptions of queer identities provided by participants]
Cavnar, 2011	USA	Qualitative	Ayahuasca	*n* = 17	43.2 (21–61)	Cis-Male (70.6%) Cis-Female (29%)	Gay (70.6%) Lesbian (17.6%) Bisexual (5.9%) Queer (*n* = 1 / 5.9%)
Cavnar, 2014	USA	Qualitative	Ayahuasca	*n* = 17	43.2 (21–61)	Cis-Male (70.6%) Cis-Female (29%)	Gay (70.6%) Lesbian (17.6%) Bisexual (5.9%) Queer (5.9%)
Ching, 2020	USA	Autoethnography	MDMA	*n* = 1	—	Cis-Male (100%)	Gay (100%)
Coveney, 2005	Ireland	Qualitative	MDMA	*n* = 46	24.3 (18–33)	Cis-Female (58.7%) Cis-Male (41.3%)	Heterosexual (91%) Gay (3%) Bisexual (3%) “Straight with bi-curious tendencies” (3%)
Denizet-Lewis, 2000	USA	Qualitative	MDMA	—	—	—	—
Dubus, 2020	France; USA	Case study; Qualitative	LSD, Mescaline	*n* = 2	17.5 (15–18)	Cis-Male (100%)	Gay (100%)
Feiner, 2019	Gorgia; USA	Theoretical; Autoethnography	MDMA	—	—	—	—
Goldpaugh, 2022	USA	Theoretical; Qualitative	All psychedelics	—	—	—	—
Jadali, 2022	USA	Qualitative	LSD	*n* = 18	—	Cis-Female (50%) Gender Non-Conforming (38.9%) Other (11.1%)	Queer (94.4%) Non-Queer (5.6%)
Klitzman, 2006	USA	Qualitative	MDMA	*n* = 12	Mean Unknown (18-45)	Cis-Male (100%)	Gay (100%)
McElrath, 2005	Northern Ireland	Qualitative; Cross-sectional	MDMA	*n* = 98	25 (17–45)	Cis-Male (69%) Cis-Female (31%)	Heterosexual (84%) Gay *or* Bisexual (16%)
Ortiz et al., 2022	USA	Theoretical	Psilocybin	—	—	—	—
Stauffer et al., 2022	USA	Qualitative; Cross-sectional	MDMA	*n* = 17	35.4 (Range Unknown)	47.1% Non-Binary 23.5% Trans Male 17.7% Trans Female 5.9% Genderqueer 5.9% Tumtum	—
Thorne, 1967	—	Case study; Qualitative	LSD	*n* = 1	43	100% Male	—

10 of the 18 publications included in the scoping review were focus group discussion or interview-based qualitative studies; 1 was a clinical trial/experimental study; 3 were case studies; 2 were theoretical/commentary pieces; and 2 were autoethnographies. Publication dates ranged from 1967 to 2022.

### Psychedelics of interest

In the extracted publications, MDMA was the most frequently-referenced psychedelic substance (7 of 18), followed by LSD (4 of 18), psilocybin (3 of 18) and ayahuasca (2 of 18). One source referred to psychedelics as a general category of substances. All but one of the LSD-focused articles were written on queer experiences in the 1960s, two of which discussed psychedelic conversion therapy. The remainder of the extracted publications were written within the last 20 years, with a shifting focus to non-LSD psychedelics such as psilocybin, ayahuasca, and MDMA (Table 2).

**Table 2:** Summary of key findings from studies.

Author & year	Study design	Sample size	Psychedelic substance(s) used/ discussed	Key findings
Agin-Liebes et al., 2021	Clinical trial; Qualitative	*n* = 9	Psilocybin	Psilocybin may enhance the effectiveness of trauma processing during adjunctive group therapy by supporting mindful awareness toward feelings, acceptance of grief and sadness, a newfound awareness of habitual protective patterns, and increasing social cohesion, trust and belonging.
Alpert, 1969	Case study; Qualitative	*n* = 1	LSD	LSD can cause changes to perceptual, cognitive, and affective organization through the dishabituation of associative processing. LSD-assisted therapy was unable to eliminate existing homosexual attraction, but was able to produce greater self-acceptance of bisexuality.
Anderson et al., 2020	Experimental	*n* = 18	Psilocybin	Psilocybin-assisted group therapy is an effective and efficient means of psychotherapy for long-term AIDS survivors who experience demoralization. Participants reported a significant improvement in mood, anxiety and existential distress.
Bryant, 2016	Qualitative	*n* = 5	All psychedelics	Experiences of non-dual consciousness, with and without psychedelics, assisted with healing LGBQ trauma and exploring identity.
Cavnar, 2011;& Cavnar, 2014	Qualitative	*n* = 17	Ayahuasca	Ayahuasca facilitated acceptance and affirmation of individuals’ sexual orientation. The reported positive effects of ayahuasca included integration of sexual identity, confidence in coming out, feelings of connectedness and naturalness and positivity to life. None of the participants experienced negative effects on their self-perception of their sexuality because of ayahuasca.
Ching, 2020	Autoethnography	*n* = 1	MDMA	MDMA-assisted psychotherapy led to increased resilience/stress tolerance, increased ability to reframe appraisals more deftly, strengthened cultural identity, increased self-compassion/self-acceptance, and increased psychological flexibility.
Coveney, 2005	Qualitative	*n* = 46	MDMA	Recreational use of MDMA increased exploration of queer sexual experiences; relaxed social norms around gender and sexuality; increased self-esteem; and improved relationships.
Denizet-Lewis, 2000	Qualitative	-	MDMA	Raves provide a safe and accepting place to explore one’s sexuality. The use of MDMA in rave settings encourages sexual experimentation and fluidity.
Dubus, 2020	Case study; Qualitative	*n* = 2	LSD, Mescaline	Psychedelic conversion therapy in France differed from similar therapies in other countries, using high doses of LSD and mescaline to induce “psychic shock.” Out of the two participants examined, neither became heterosexual.
Feiner, 2019	Theoretical; Autoethnography	-	MDMA	The use of MDMA in rave settings helps to break down boundaries between racial groups and between different sexual identities. Queerness and MDMA are vilified as part of a larger campaign against raves and countercultural movements.
Goldpaugh, 2022	Theoretical; Qualitative	-	All psychedelics	When properly supported and integrated, psychedelic integration therapy may produce sustained trauma healing, increased sexual satisfaction, and well-being. Profound experiences of embodied pleasure, communication with the divine, and letting go of shame during psychedelic experiences can help resolve sexual trauma.
Jadali, 2022	Qualitative	*n* = 18	LSD	LSD may be used as a mechanism for spiritual exploration, shaping understandings of the self, suspending social mores, and working through conflicting identities that may prevent spiritual exploration.
Klitzman, 2006	Qualitative	*n* = 12	MDMA	MDMA use can break down social barriers among gay men and increase social bonding. Participants reported MDMA’s utility in countering feelings of stigmatization and loneliness. Participants felt closer to others, a greater sense of community, camaraderie and connection.
McElrath, 2005	Qualitative; Cross-sectional	*n* = 98	MDMA	Sexual behavior while under the influence of MDMA differed between participants. Some female participants described being more open to exploring bisexuality while on MDMA. Gay and bisexual women were more likely to use MDMA as a sexual enhancement. The primary motivation for MDMA use was to experience an increase in sensuality and intimacy.
Ortiz et al., 2022	Theoretical	-	Psilocybin	Vulnerable populations have a complex history with psychedelic-facilitated psychotherapy. Vulnerable populations have additional areas of concern that must be considered in future research practices. Sexual orientation is routinely not reported in clinical trials with psilocybin.
Stauffer et al., 2022	Qualitative; Cross-sectional	*n* = 17	MDMA	Transgender and gender-diverse people face unique challenges when accessing trauma-related mental health support that must be considered for MDMA-assisted psychotherapy. Adaptations such as gender-inclusive intake procedures, gender-affirming providers, diverse care teams and options for group therapy may increase access to MDMA-assisted psychotherapy for transgender and gender-diverse populations.
Thorne, 1967	Case study; Qualitative	*n* = 1	LSD	Marital LSD therapy may increase self-acceptance, improve interpersonal relationships, heal from family trauma, and result in a positive spiritual experience.

## Discussion

### Who is being studied?

A defining strength of the queer community is the diverse, ever-evolving, and ever-expanding possibilities of self-identity with which one can align and express. Unfortunately, this heterogeneity and richness is not reflected in the queer psychedelic literature.

Gender-wise, the lived experiences of gay cisgender men dominate the narrative of the included resources. Of the studies screened, only five reported an explicit breakdown of participants’ gender that includes data beyond “cisgender gay man,” and only three of these studies account for genders beyond the cis-binary (i.e., genderqueer, non-binary, etc.). To this point, we wish to note that there was a large proportion of studies focused on the gay cis-male experience that we excluded for adopting a pathologizing lens on psychedelic use. The most recurrent trend among these studies were investigations into the prevalence of use of MDMA among gay men in the rave scene in the 1980s and 90s with the conclusions being that gay men used MDMA in greater numbers than heterosexual men (38). The studies perpetuated the ‘othering’ of the gay community at best and promoted outright homophobia at worst. Regardless of whether the lens was supportive or pathologizing, similar to many other fields of research, the cis-male experience has been highly overrepresented in the literature.

In comparison, we did not locate any studies that focused specifically on the lived experiences of queer womyn or female-presenting psychedelic users. Discrimination can occur through commission or through omission. For example, in early literature on queer psychedelic use (39), it is mentioned that a cis-male subject’s wife exhibited bisexual tendencies, however her lived experience was completely erased from the study. As we have seen in recent years through the work of Caroline Criado-Perez (40) for example, the experience of womyn and female-presenting people have been systematically and pervasively under-researched in many fields, often with detrimental and sometimes life-threatening consequences. This is a vast lacuna in the literature that must be addressed moving forward. Thus, while this scoping review represents a call to deepen our understanding of the queer experience in psychedelic studies, it is essential that this call to action be understood as both quantitative (calling for more data) and qualitative (calling for more inclusive research) in nature.

The issues faced by lensing the research along the gender binary is also reflected in the lack of diversity represented along the spectrum of sexual orientation. Of the studies screened, six were unclear about what sexual orientations were included as part of the study, or provided no sexual orientation breakdown beyond their research speaking to the experiences within the queer community broadly. We also observed issues in the literature conflating transness with sexual orientation, or potentially, with “transvestitism” (39), a stigmatizing term no longer used today that prevents modern researchers from knowing the extent to which actual gender identity was being considered. In the resources screened in, if the varied sexual orientations of participants were discussed at all, most were lumped in as ‘queerness’ or were lensed through the gender binary (i.e., gay, lesbian).

As it stands, heteronormative bias is pervasive throughout the existing psychedelic literature and continues to be a substantial barrier to queer inclusion. Researchers perpetuate heteronormative bias and center the experiences of cis-heterosexual participants through methodological shortcomings such as ineffective or an absence of reporting on sexual orientation and gender identity (41), inappropriate categorization of queer individuals (42), an emphasis on the male/female therapist dyad (43), and a lack of community-based and intersectional methods (44). Ineffective, inappropriate or missing demographic data on queer identity removes the unique insights offered by queer people and shows apathy and disinterest in studying queer populations (42, 44). Elements of study design that reinforce the gender binary like the male/female therapist dyad, may disenfranchise queer, particularly transgender, participants (43). Failure to utilize methodologies that seek to include queer perspectives actively perpetuates heteronormative bias by either assuming the participant is cis-heterosexual or excluding queer participants by creating barriers within recruitment or study design.

That said, modern resources included in the review point toward a current shift in inclusivity of gender and sexuality. Jadali (45) explored how non-binary Islamic youth understand, conceptualize, and relate to God through psychedelics. Stauffer et al. (46) described insights from transgender and gender diverse focus group discussants about queering the frontiers of PTSD treatment trials. Additionally, Bryant’s (47) study on psychedelic experiences of non-duality and sexual identity provided participants with an open-ended opportunity to self-identify both in terms of gender and sexual orientation, and collected that data as a person’s ‘current sexual identity,’ allowing for the fluidity of identity. Coveney (48) also took an inclusive, open-ended approach to sexual orientation self-identification, rather than making study participants choose from a predetermined list of “gender and sexual orientation typologies.” These inclusive data collection methodologies, while encouraging, have not been reflected in the majority of studies conducted since 2005.

Finally, we note that there is a distinct lack of consideration paid to other intersectional and minoritized identities of queer participants. When it comes to race, for example, 10 studies provide no racial breakdown or assume ‘white’ as the default, while six studies only provide some basic information on the racial breakdown of participants. Only three studies included an attempt at analysis and discussion through a racial lens. As the field expands, in addition to broadening the range of gender identities and sexual orientations studied, there is a need to capture the experiences of a wide range of minoritized queer experiences.

### The need for intersectionality

Intersectional approaches to research account for how an individual’s various identity factors (gender, sexuality, ethnicity, class, religion, disability, etc.) intersect and overlap to create novel lived experiences of privilege and oppression (49). Intersectional researchers thus acknowledge that queer identities never exist in isolation and know that what is true of one queer person’s experience will rarely be true for the whole queer community (e.g., a queer woman of color may face discrimination not only based on her sexual orientation, but also on her gender and racial identity—experiences to which queer white men may struggle to relate).

By incorporating intersectionality into queer research, scholars and clinicians can better understand the diverse experiences within the queer community. This helps to recognize and address the specific needs, challenges, and inequalities faced by individuals who exist at the intersections of multiple marginalized identities. Failing to account for these intersections when conducting research on queer populations tends to produce reductive research that fails to capture the lived experiences of all of those being studied, or worse, researchers completely neglect to include certain demographics in their research (e.g., trans, disabled, or non-white queer participants), which limits the generalizability of their findings—as observed in Anderson et al. (50). Furthermore, neglecting intersectionality in clinical settings can lead to missed psychosocial and cultural components of illness presentation and worse rapport with treatment providers (51, 52).

While the intersections of gender and sexual orientation are discussed to some extent in the majority of applied research publications reviewed, authors often neglected to collect detailed data on participant ethnicity, class, religion, or ability. In some studies, this demographic data *was* collected, but analysis of how these other identity factors interfaced with participants’ queer identity was limited or non-existent [e.g., (45, 47, 49)]. Recently, however, new publications involving demographically homogeneous participant groups explicitly recognize how this limits the generalizability of their findings, potentially reflecting a growing awareness among researchers regarding the importance of diverse sampling and intersectional approaches in applied research [e.g., (45, 52)]. In some cases, authors explicitly named the need for more intersectional approaches in queer and/or psychedelic research (41).

Only two applied research studies took an explicitly intersectional approach. Ching’s (53) auto-ethnographic report of his experience as a participant in a clinical trial for MDMA-assisted psychotherapy highlights the therapeutic implications of intersectional insights in psychedelic-assisted care involving racialized queer individuals. Jidali’s (45) qualitative study of 18 queer/trans Iranian Americans is also highly intersectional in its consideration of how racial/ethnic, gender, sexual, religious, and class identities compound to create unique lived experiences of privilege and oppression among study participants, though this consideration is limited when discussing these individuals’ psychedelic experiences. Both Cavnar (54, 55)^2^ and Bryant (47) exhibited a degree of intersectionality in that study participants’ experiences were analyzed with consideration for how their gender and sexual identities interfaced with their spiritual or religious backgrounds. That said, how participants’ queerness interfaces with other identity factors such as class, race/ethnicity, or disability receives limited or no attention.

Overall, the queer psychedelic literature included in this review does not perform well with intentional, intersectional lensing of queer psychedelic experiences, often focusing on single identity parameters (e.g., sexual orientation only or gender only) or not integratively considering psychedelic experiences within the container of diverse, interacting, and intersectional identities (i.e., gender *and* sexual orientation *and* race, etc.). This aligns with claims made elsewhere that despite growing recognition of the importance of intersectional approaches to both queer research and psychedelic research (1), intersectional theory remains poorly understood and is rarely deployed effectively in applied research (56). There does, however, appear to be a growing recognition of the need for more inclusive and intersectional research approaches in the study of queerness and psychedelics, a trend in the literature which seems to have gained popularity since 2020.

An important takeaway in reviewing the intersection of psychedelics and queer identity has been the lack of literature on the subject. While studies have recently made increased efforts to capture data on sexuality and gender, little research has been done to specifically explore the unique ways in which queer individuals may benefit from psychedelics, and almost no psychedelic research has explored the intersectionality of queerness and other identities. Currently psychedelic research is primarily funded with the aim of treating psychiatric and other illnesses; however, more research is needed to explore how identity both shapes and is shaped by these substances.

### Varied motivations for use

It is easy today to draw similarities between psychedelic use and sexual identity. The exploration of consciousness and the pursuit to understand oneself and one’s place in the world is a persistent theme in both queer studies and psychedelic research. The ability to induce transformative experiences that impact a sense of identity, self-acceptance, and connection to others is central to the intersection of queer identity and psychedelic use. Understanding the historical context of both queer identity and psychedelic use is crucial however for contextualizing their intersection and the dramatic changes that have occurred underlying motivation to use psychedelics within the queer community.

As the 1950s and 1960s witnessed a surge in interest in Western culture around the therapeutic potential of psychedelics for anxiety and alcohol use disorder, some practitioners, including psychedelic “gurus” Leary, Alpert, and Grof, incorporated psychedelics into their conversion therapy practices (57). Psychiatrists used LSD and mescaline as “psychic shock” therapy, and the original transformative goals in regards to the queer identity were not self-acceptance, but instead radical self-denial. Queer individuals either sought or were forced into intervention to fit within the framework of socially acceptable sexual practices. The clinical motivations behind implementation of psychedelic conversion therapy were laid explicit by psychiatrist Thomas Szasz in saying “it is a question for therapists of whether they are defending the personal autonomy or the social control of deviants” [(56), p. 653].

In Dubus (57), the authors describe how adolescents, at the peak of identity realization, could be prosecuted and admitted to psychiatric units for treatment of “immodest or unnatural acts.” While under the influence of psychedelics, therapeutic goals placed emphasis on the pathologization of their queer identity, such that describing queer aspects of their identity was labeled “filthy” and could even lead to being placed in restraints. Not uncommonly patients were encouraged to engage in sexual activity with the opposite gender while their responses were psychoanalyzed (58). Even in patients who sought out these therapies, such as the one discussed in Alpert’s (58) case report, the goals of the therapy in treating queerness as a diagnosis inherently stripped patients of self-actualization.

As psychedelics fell out of use in Western medicine and research by the 1970s due to criminalization and the War on Drugs (59), and queer identities were eventually no longer pathologized by mainstream medicine (60), the intersection of queerness and psychedelics seemed to shift. Queer individuals began to use psychedelics not to conform to societal norms, but rather to break through them. Beginning largely in the 1990’s raves began to replace the discotheques as safe spaces for queer individuals to find acceptance despite persecution by mainstream society (61). With the rise of rave parties, MDMA use, which was previously limited to big city clubs and college campuses, began to become more prominent in the community. For example, in a feature piece in *The Advocate*, (62) describes how queer individuals—especially teenagers—sought out MDMA at raves. Using MDMA in the rave scene facilitated greater feelings of acceptance and inclusivity in the queer community. Individuals were able to experiment openly due to the “breakdown of inhibitions” that occur on MDMA, and in doing so experienced a sense of empowerment through psychedelics that had not previously been accessible—also discussed in Coveney (48).

As psychedelics continue to regain acceptance in Western medicine, greater emphasis has been placed on the importance of set, setting, and intention (63). Psychedelic use that was once condemned as problematic in rave scenes and counter-culture, is now promoted as therapy to help navigate conflicts of self. One of our screened-in studies of ayahuasca use by gay and lesbian identifying individuals, for example, found that people reported participation in ayahuasca ceremonies helped remove barriers to self-acceptance of one’s sexual identity (54, 55). Additionally, psilocybin has been used to treat feelings of demoralization and social isolation in long-term HIV survivors (50), and MDMA has been proposed as a possible therapeutic intervention to help transgender and gender diverse individuals suffering from PTSD (46). The therapies today are inherently self-actualizing, as the goal is no longer to “correct deviant behavior” but to help individuals embrace their full identity. As we continue to consider the medicalization of psychedelics in regards to queer identity, clinicians should empower clients to be active participants in creating set, setting, and intention that is affirming of their individual identity. By collaborating together on the goals of treatment of psychedelic psychotherapy, queer individuals may better address their mental health needs without fear of pathologization of their identity.

### Varied settings of use

While not foreseen as a potentially relevant avenue of inquiry in our original research questions, the question of setting emerged as an interesting point of reflection and analysis. In the extracted articles, while psychedelics were consumed in diverse settings, there seemed to be a fairly even split between the consumption of psychedelics individually—either alone or as accompanied by supportive figures—or in the presence of others also partaking in the psychedelic experience (i.e., group psychedelic experiences). For example, case reports described individual or marital LSD-assisted conversion therapy in a clinical setting [e.g., (39, 57)], as well as individual MDMA-assisted therapy promoting identity affirmation in a controlled setting (53). Other studies described how certain individuals may consume psychedelics alone in nature in order to promote closeness to God (45). Some examined the consumption of various psychedelics in the presence of like-minded communities for fun/pleasure [e.g., (46, 63, 81)], or for the express purpose of healing or spiritual growth from past adversity [e.g., (46, 54)]. Yet other research showed how individual psychedelic use in controlled settings with therapists could be blended with and supported by group therapy sessions, as in the open-label psilocybin study by Anderson et al. (50) for demoralization among long-term AIDS survivor men.

While we discuss outcomes of psychedelic use below, a noteworthy emergent observation was that settings of psychedelic use may unpredictably reflect or predict outcomes of use. For example, queer individuals may derive immense pleasure from recreational group psychedelic experiences as such experiences in a communal setting have the potential to catalyze healing for queer stress and trauma—e.g., in Bryant (47). Likewise, individual psychedelic use in therapeutic, controlled settings may also be a source of objectively hedonistic experiences [e.g., (64)]. This highlights the reality and power of queer spaces to shape queer experiences, which may be amplified with psychedelic use. Communal spaces such as queer clubs, bars, or rave scenes that outwardly signal a permissiveness for queer joy and expression likely also facilitate a process for healing from queer stress and trauma, whether or not participants are in a chemically altered state (48, 65–67, 81).

### Outcomes and impacts of queer psychedelic use

Very few outcomes and impacts of queer psychedelic use have been documented as part of experimental research due to the dearth of clinical studies investigating this topic. The present study’s database searches suggest that Anderson et al. (50) is the only such study conducted to date. Empirical findings of the therapeutic potential of psychedelic-assisted therapy specifically among queer populations thus remains limited. Furthermore, this pilot study presents a very small and homogenous data set of exclusively gay-identified, English-speaking, cis-gender male AIDS survivors above the age of 50, most of whom were white, so the generalizability of this study’s findings is limited. Nonetheless, the outcomes are promising for the potential of psychedelic-assisted therapy in queer populations. This study employed a novel group-therapy model (12–15 h over 8–10 meetings) combined with a single moderate-to-high dose (0.3–0.36 mg/kg) 8-h individual psilocybin-assisted therapy session. No serious adverse reactions to psilocybin were observed, and at 3-month follow-up, investigators detected a clinically significant reduction in demoralization, measures of self-reported PTSD, and complicated grief, which were assessed using the Demoralization Scale-II (DS-II), PTSD Checklist-5 (PCL-5), and Inventory of Complicated Grief-Revised (ICG-R), respectively. While the demoralization and complicated grief measures were used to track participants attitudes related to living with AIDS and surviving the AIDS crisis, the finding that psilocybin-assisted group therapy may help in the treatment of trauma-related illness is significant for the broader queer community, as queer individuals experience trauma-related illness at considerably higher rates than their cisgender/heterosexual peers (68), and there is a need for innovative treatment approaches to trauma-related illness in this population. Further research into the therapeutic potential of psilocybin-assisted trauma therapy is thus warranted, with particular attention given to the potential value of group-based interventions, as both Agin-Liebes et al. (64) and Bryant (47) remark upon the value of queer individuals doing psychedelic-assisted trauma-processing with others who have a shared history of adverse life experiences.

Though not stated explicitly in the screened-in publications, one theme present throughout this review is the use of psychedelics to combat minority stress. The minority stress described in this review includes homophobia, sexual trauma, social stigma around sexual orientation and gender, and HIV-related trauma. A common conclusion drawn by several of the studies is that psychedelics help process this trauma through self-affirmation and acceptance. Queer trauma, and minority stress in general, is due in part to the isolation and lack of social capital experienced while living in a homophobic society (66). Often the focus of queer trauma in the medical field relates to HIV stigma as discussed by Anderson et al. (50) and Agin-Liebes et al. (64), yet feelings of shame, exclusion, and fear for safety are very common across queer individuals (69, 70). As a potential balm for this wounding, psychedelic healing often promotes feelings of connection (71, 72). The feelings of being connected to nature, life, people, and one’s body and sense of self can help break the patterns of learned hatred that society has reinforced in queer individuals. Inherent in connection is acceptance, and to feel a sense of belonging, sometimes for the first time, can be a radical worldview-shifting experience for those who have existed on the margins of society.

The articles included in the present scoping review also suggest that another major outcome of psychedelic use among queer individuals is a deepening of religious or spiritual attitudes. This is significant because the spiritual or religious lives of queer individuals are often encumbered with the fact that many religious doctrines have traditionally regarded queer relationships or identities as inherently sinful or immoral (47, 54). Because of their immediacy and relative independence from cultural systems of power and oppression, psychedelic experiences may provide queer individuals a container to explore their spiritualities that are notably safer than other, more institutionally-bound religious containers. The experiences described in the reviewed publications varied considerably, with some queer individuals using psychedelics to connect with higher dimensions of reality, cultivate a “sacred connection to nature,” and come to grips with their mortality [(46), pp. 109, 139]. Thorne (39) mentioned an individual attempting LSD-assisted conversion therapy experiencing a “total oneness with God” where “he saw himself as a piece of thread in the whole pattern of the universe” (p. 174). Cavnar (54) additionally described ayahuasca users “opening to spirit and higher realms of consciousness” and being given a cosmic “mission” from God—imbuing their time on Earth with abiding purpose (pp. 56, 66). Jadali (45) too described how LSD served to help several queer Iranian youths establish a direct connection with God, and reconceptualize the Divine as an all-encompassing, nonhierarchical web of interconnected energy binding and manifesting as all living things. In all of these cases, psychedelic-assisted deepening of spiritual attitudes directly facilitated emotional breakthroughs which promoted increased self-awareness and self-acceptance [e.g., (39, 46, 53)]. In a world where spiritual resourcing and support happens largely within religious communities with a history of queer- and trans-phobia, queer “seekers” may experience psychedelic spirituality as a profoundly liberating avenue for self-exploration and spiritual transformation.

### The psychedelic experience as queer identity development

A salient finding that emerged in the literature was the spectrum of queer identity development processes that mapped on to participants’ psychedelic experiences. For example, Coveney’s (48) albeit predominantly heterosexual sample described various impacts of the acute effects of MDMA on gender and sexual expression. Specifically, the inherent ‘queerness’ of “ecstasy culture” provides a permissive premise for exploration of one’s gender and sexual identity, even if already established (i.e., as heterosexual). Notably, Coveney (48) explicitly discussed how because of the “breakdown of inhibitions” with MDMA, one user in particular experienced greater sexual fluidity recognizing their attraction to individuals of the same gender (pp. 223-227). Cavnar (54, 55) provided further convincing evidence of psychedelic-catalyzed queer awakenings with their sample of queer ayahuasca ritual participants. In that study, some participants described receiving inspiration to ‘come out’ as self-accepting/−affirming gay or lesbian individuals despite foreseeable challenges in their lives after the ceremony. Some of these participants also described motivation to remove barriers to living more fully in their gay and lesbian identity post-ceremony.

The above examples signal a move along the queer identity development spectrum from exploration to self-acceptance and self-affirmation. In Cavnar (54, 55) as well as Goldpaugh’s (73) opinion piece, there is the notion that not only can psychedelics awaken sexual identity through affirmative processes, they can also promote sexual healing, functioning, thriving, and reclamation of the self as a sexual being. Ching (53) provided further evidence of the queer-affirming outcomes of a profound MDMA experience, with a vivid narration of the highly visual process of unburdening and unshackling of intersectional minority stress as a cisgender, gay, Singaporean Chinese, immigrant man during the dosing session. Ching (53) described leaving the experience confident and self-efficacious about navigating a racist and heteronormative society as a fully affirmed gay Asian man.

Moving along the spectrum, we encounter the prospects and reality of queer identity fatigue, this time among long-term survivors of the HIV/AIDS epidemic and governmental denialism in the United States (50). In Anderson et al.’s trial (50), psilocybin combined with psychological support in a hybrid individual-and-group format was safe and efficacious in addressing demoralization, and feelings toward experiences of isolation and social stigma. In several participants the authors discuss a psilocybin-catalyzed reaffirmation of oneself as a proud queer individual, in spite of earlier-life adversity and minority stress and trauma.

The feelings of connection provided by psychedelic experiences can offer the support needed to open oneself to self-reflection and self-acceptance. It is this state of vulnerability that Goldpaugh (73) describes as facilitating self-acceptance, sexual fulfillment, trauma processing, and enhanced creativity. Through the breakdown of the ego—the sense of self born from interacting with society and environment—a new sense of self, created and embraced by the individual, has a chance to emerge. In many ways, opening up to this new sense of identity has parallels with the experience of coming out of the closet for queer individuals. The bits and pieces that make up one’s identity were always present, but by finally putting them together, one’s view of oneself is forever shifted. Furthermore, similar to the importance of safety in the process of coming out, the setting in which one puts these pieces together greatly influences the end result. It is for this reason that set and setting tailored to queer individuals and in consideration of the queer experience is vital to psychedelic social, spiritual, and personal empowerment.

As a final observation, Alpert’s 1969 case study described a 38-year old bisexual man seeking LSD-assisted “therapy” to eliminate his attraction to men (58). While he had had sexual encounters with womyn prior to the intervention, he found these experiences generally unsatisfying and predominantly gravitated to homosexual experiences instead. Alpert claimed that through psychedelic-assisted “perceptual-cognitive-affective reorganization” (a loosening of habitual associations), the bisexual man in the study could potentially overcome some of the psychological barriers he experienced with regards to opposite-sex attraction. In some sense, this intervention worked in that the participant found his romantic relationships with womyn to be more intuitive and rewarding in the months after the intervention, but importantly, the participant continued to experience same-sex attraction. This suggests that while psychedelics may help individuals fully express their sexual identities by exploring repressed sexual attraction, they are unlikely to eliminate pre-existing sexual attraction. This mirrors the “breakdown of inhibitions” seen among self-described heterosexuals in Coveney (48) using psychedelics to explore repressed homosexual attraction, while in Alpert (58), psychedelics aided in the breakdown of heterosexual inhibitions in a bisexual man. Despite psychedelics being used to facilitate heterosexual attraction in this example, the identity development should still be interpreted as queer, as the participant was able to more fully embrace/manifest his bisexual identity through his psychedelic experiences.

## Limitations

The present scoping review has several limitations. First, attention must be drawn to the fact that despite the number of databases searched and the extensive list of keywords used in those searches, there is no guarantee that all possible publications meeting this scoping review’s inclusion criteria were captured. There are likely other resources that were not captured despite our effort to be as broad and inclusive as possible with our search terms and databases used. In a similar vein, the authors recognize that research on the intersections of queerness and psychedelics is a relatively new and quickly evolving topic. Therefore, in addition to missing some key resources within the catchment dates, there may be several publications discussing queerness and psychedelics that may have been screened in since our most recent database search (conducted in May 2023) and the publication of this scoping review. Notably, this extends to the exclusion of gray literature from this review. Gray literature was omitted for the present study primarily due to the restrictions of the academic search databases used. However, gray literature makes up the majority of publications on the intersections of psychedelics and queerness. This may indicate a relative lack of interest in or funding for queer psychedelic research projects within scholarly institutions, or it may be a byproduct of queer theorists taking a deliberately emic approach to queer research where queer communities can tell their own stories. The present scoping review can therefore not be considered comprehensive, and a full exposition of the discourse on psychedelics and queerness would likely require a complementary review on gray literature.

Some of the gray literature not captured in our search includes (but is not limited to): the Chacruna Queer Archives, a collection of online essays exploring a wide range of topics involving psychedelics and queerness (74); an auto-ethnographic report of how LSD experiences can affect gender dysphoria among trans individuals (75); several MAPS Bulletin articles addressing queer issues in psychedelia [e.g., (75–77)]; a chapter from Pienaar et al. in *Cultures of Intoxication* discussing how drugs like LSD and MDMA can help trans individuals “remove a lot of gender dysphoria” and unpack traumas associated with one’s marginalized gender and sexual identities [(78), pp. 149-154]; and two major collections of essays from Chacruna: *Psychedelic Justice: Toward a Diverse and Equitable Psychedelic Culture* (79) which has a five-essay section on psychedelics and queerness; and *Queering Psychedelics: From Oppression to Liberation in Psychedelic Medicine* (23), the first ever full-length book dedicated to discussing the psychedelics-queerness intersection.

Topics covered in *Psychedelic Justice* (79) and *Queering Psychedelics* (23) include the history of psychedelic conversion therapy, how to co-create queer-affirming psychedelic treatment practices, using intersectionality-informed liberatory tools in psychedelic therapy with queer individuals, and the promise of psychedelics for promoting queer pleasure and identity development. These gray literature sources represent a stronghold in the discourse where the potential benefits of queer identity affirmation through psychedelic experiences are explored freely, unfettered by the traditional pathologizing of queerness or drug use seen in mainstream academic literature.

This list of gray literature sources is by no means exhaustive, but it is clear from the number of publications mentioned here that research on psychedelics and queerness outside academic institutions is outpacing research taking place within them. This may indicate a relative lack of interest in or funding for queer psychedelic research projects within scholarly institutions, or it may be a byproduct of queer-theorists taking a deliberately emic approach to queer research where queer communities can tell their own stories, unhampered by the patriarchal and colonial notions of scientific legitimacy that dictate what scholarship is or is not publishable within Western academia [(80), p. 146].

Lastly, a discussion of methodological challenges associated with the inclusion and exclusion criteria of the present study is warranted—namely, the authors recognize that despite their efforts to ensure inter-rater reliability and maintain definitional consistency throughout the reviewing process, what constitutes a ‘meaningful’ or ‘stigmatizing’ discussion of certain topics is open to interpretation. Gender and sexual orientation are inherently subjective, and thus a qualitative analysis of the discourse on such topics will inevitably reflect to some degree the authors’ biases despite attempts at inclusivity and objective observation. The biases of each author’s perspectives were minimized using the consensus decision-making approach described above, but other reviewing teams may have produced varied results. The authors encourage readers to examine their own definitions of ‘meaningful’ discussion and ‘stigmatizing’ perspectives when considering the conclusions drawn in the present study.

## Implications and recommendations

There is an undeniable and significant opportunity to increase both the quality and quantity of research into the meaningful overlap of queerness and psychedelic experiencing. Below is a summary of some of the key takeaways and recommendations:

### More queer research needed

As evidenced by the outcomes of this scoping review, there is a clear gap in the psychedelic studies literature, and it is significant that the publications that were excluded for being overtly pathologizing to the queer community outnumbered those that were screened in. Addressing the gap in the peer-reviewed literature on psychedelics and queerness also requires not just collecting better data, but also disaggregating and analyzing the data collected to discuss findings about queer psychedelic users and draw meaningful conclusions about this intersection. As outlined in this paper, very little research has been done to specifically explore the unique ways in which queer individuals use, experience and may benefit from psychedelics. To that end, there is a need to specifically research the queer psychedelic experience, not just as a subset of a larger data set, but also by developing standalone studies (whether conducted by academics or laypersons) with non-stigmatizing research questions that directly inquire into the motivations, impact and lived experience of psychedelic use by queer individuals.

Psychedelic researchers might also consider implementing more inclusive data collection methods, such as the open-ended self-identification of gender and sexual orientation suggested by others. The value of peer-reviewed autoethnographic articles examining psychedelics and queerness should also be emphasized, as they help bridge the gap between traditional academic research and the world of Queering Psychedelics gray literature. This is significant, as self-reported anecdotal evidence can provide highly detailed qualitative data relating to the unique therapeutic potential of psychedelics for queer populations. Promoting this methodology in peer-reviewed psychedelic research would help provide better frameworks for facilitating queer-focused psychedelic therapy sessions. As an empirical methodological approach, we might also envision a large-scale survey of psychedelic use in queer individuals with questions tied to the phenomenology of queer psychedelic use that we have uncovered in this scoping review. For instance, to what extent do queer individuals attribute positive identity development to past psychedelic use? Or, to what extent do queer individuals agree that psychedelic use in queer settings are constitutive of a safe space?

### More queer researchers needed

The queer psychedelic research gap will continue to be filled by queer psychedelic researchers and queer-friendly psychedelic publishers who prioritize and advocate for the need to better understand this understudied topic. However, the authors of this scoping review (who are all members of the queer community) would also like to call on the hetero and cis-male-identifying communities to step up as allies to help queer the psychedelic space. Like other calls for allyship among marginalized communities, privileged identities can help create conditions for marginalized voices to be heard. For example, hetero, cis-male-identifying people amplifying or deferring to voices that have been silenced or marginalized within the psychedelic movement. Leaders of institutions, organizations, and companies might also consider using funds and resources to create spaces and opportunities to discuss and highlight queer issues. And finally, ensuring appropriate compensation and recognition of queer researchers (especially those with intersecting marginalized identities) laboriously fighting against societal transphobia and homophobia would promote the sustainability of this research.

### More research informed by the unique lived experience and wisdom of queer people is needed

As highlighted in the discussion of this study, the inclusion of queer lived experience is not just of benefit to the queer community (which has so often been marginalized in the psychedelic research space). By deepening our understanding of the queer experience and some of its unique features, we can also benefit and help deepen our understanding of the broader human-psychedelic experience. As discussed, some of these core questions might include:

- Identity: Exploring one’s identity and understanding of self is fundamental to psychedelic experiencing, and also to being queer. One of the greatest strengths of queer culture and community is the variety of ways that people can identify and express their authentic selves. This grappling with, and openness to, a wide range of self-understanding outside the ‘or’ of the binary, also highlights the ‘and-ness’ of the queer experience. Being queer is inherently a creative act. For ultimately, being queer is not just about pronouns or who a person has sex with. Being queer involves intentionally grappling with questions of how to give and receive love, how we love each other and love ourselves. These are some of the fundamental questions that people often have to confront in psychedelic experience.
- Community: While much research is being conducted on the therapeutic use of psychedelics in medicalized settings, there is a small but growing conversation around the importance of community and peer support in psychedelic experiencing—an approach that queer folks have known about for a long time. Communal experiences such as ‘found family’ and safe spaces such as queer clubs, bars, or rave scenes that outwardly signal a permissiveness for queer joy and expression have long been shown to facilitate healing from queer stress and trauma. Thus, while there is a need to better understand the therapeutic use of psychedelics to combat queer trauma, there is wisdom within the queer experience to help inform a more comprehensive understanding of the potential of a range of therapeutic psychedelic sets and settings, which can be of benefit to a wide variety of marginalized groups and lived experiences. We also point to the opportunity of leveraging the community orientation of queer healing and gathering as a solid jumping-off point for better understanding the potential of group psychedelic and integration work. This should include further research investigating the potential of psychedelic-assisted group therapy models.
- More diverse research needed: The quantity of queer psychedelic research needs to be improved, particularly clinical research on the therapeutic potential of psychedelic-assisted therapies for queer populations. A greater number of experimental studies producing empirical, quantitative data would help demonstrate the safety and efficacy of this therapeutic intervention for queer populations. The quality and diversity of that research also warrants attention. This review’s findings also suggest the psychedelic research community needs to build a more inclusive understanding of the experiences of (a) queer womyn and womyn-presenting people; (b) a wider range of queer sexual orientations and identities; and (c) other intersecting identities such as race and ability. As evidenced in the literature, there is a strong orientation toward the cis-gay-male experience, resulting in the invisibility of womyn and female-presenting folks, but also exacerbating the invisibility of non-binary and bisexual people, as well as trans, pansexual, two spirit, asexual and other queer identities and ranges of lived experience. For that reason, researchers should reference literature that describes safe, effective and inclusive methodologies for queer populations. Implementing practices such as having a diverse research team, prioritizing transparency and safety, recognizing intersectionality, acknowledging the influence of minority stress and using effective recruitment strategies such as utilizing community partners, accessible language, and liberalizing inclusion criteria are all ways to make psychedelic research more accessible to queer people. Social and systemic discrimination has dire consequences for all marginalized groups, including queer individuals. Marginalized people are often those most in need of support, healing and community, and yet are also the ones most often omitted from the psychedelic research space. The impacts of systemic discrimination are well understood and can help us understand why there is such a lack of research into the queer experience. However, this lack of gender and sexual diversity (as well as ability and racial diversity) in psychedelic research and in clinical trials may come from a wariness that many oppressed groups have toward participating in medicalized and non-medicalized spaces which have often ignored and stigmatized them. For example, gender dysphoria and transphobia can often prevent transgender and non-binary people from entering often-gender-segregated ayahuasca circles; and the history of using psychedelics for ‘conversion therapy’ has been well documented. That often-unacknowledged legacy may contribute to keeping queer people away from participating in research trials. Therefore, as the psychedelic research community works to build a more inclusive engagement with the queer community, there is an urgent and concurrent consideration toward the acknowledgement of past and present harms, and the development of safe spaces and practices in order for queer people and all marginalized identities to feel able to actively engage.

## Conclusion

Research has shown that psychedelics have the potential to enhance meaning and creativity, providing new insight and experience that can be personally, psychologically, and spiritually significant for many different people. We contend that this significance may be especially true for queer individuals. As shown in this scoping review, however, this significance remains underexplored in peer-reviewed academic research. From our findings, it is clear that there is a strong need for broader and more inclusive research investigating the intersections of queerness and psychedelic experiences, as well as the therapeutic potential of psychedelic-assisted therapies among queer populations. As we have explored in the discussion of this paper, queerness itself is a form of embodied creativity, and the fact that queer individuals exist is creativity in action. It takes ingenuity to move from surviving to thriving in a heterosexist world, and it takes tenacity to create queer spaces that legitimize, support and celebrate our existence. Inherent in being queer is also a commitment to understanding love—finding love and compassion for oneself, and working to access love for and with others. Queerness is a process of learning to experience love as acceptance, love as a daily practice, and as often experienced in psychedelia, love as both an act of creativity and an act of creation. By reclaiming, redefining, and reimagining the meaningful relationship between psychedelics and the queer experience, we hope this review helps move the research and clinical conversation in a queerer direction, centering queerness and queer experiences as an essential component of psychedelic research and practice.

## Data Availability

The original contributions presented in the study are included in the article/supplementary material, further inquiries can be directed to the corresponding author.

## Footnotes

^1^In this scoping review, we use the reclaimed term “queer” as an inclusive umbrella label to encompass a wide range of identities, including lesbian, gay, bisexual, transgender, intersex, asexual, two-spirit, and others (LGBTQIA2S+). We recognize that language is complex and that the term ‘queer’ may not resonate with everyone. However, we have chosen to use this term for its potential to foster both inclusivity and efficiency in our discussions. Our aim is to honor the diverse range of choice and experience within the community and by choosing a term that is meant to be inclusive, the term is also meant to acknowledge that identities are subjective, fluid and evolving.

^2^Both these publications were authored by the same person and concerned the same sample of 17 study participants.
